# Tyrosinase Small Interfering RNA Effectively Suppresses Tyrosinase Gene Expression In Vitro and In Vivo

**DOI:** 10.4061/2010/240472

**Published:** 2010-11-04

**Authors:** Jia Xiu-Hua, Lin Shao-Chun, Huang Bing, Zhu Xiang, Zhuang Jing, Li Wei-Hua, Liu Qian, Luo Ting, Xu Xiao-Ping, Chen Xi-Gu

**Affiliations:** ^1^State Key Laboratory of Ophthalmology, Zhongshan Ophthalmic Center, Sun Yat-sen University, Guangzhou 510060, China; ^2^Central Laboratory, The Third Affiliated Hospital of Sun Yat-sen University, Guangzhou 510630, China

## Abstract

Tyrosinase is a bifunctional enzyme which oxidizes the initial step of melanin biosynthesis, that is, conversion of tyrosine to dopa and subsequently dopa to dopaquinone. It is a glycosylated protein and a major regulator of melanogenesis. To date, many approaches have been tried to regulate tyrosinase activity and melanin content. To that end, we screened small interfering RNA sequences for sequence-inhibited tyrosinase expression in B16 cells and in C57BL/6 mice. We analyzed tyrosinase mRNA levels by quantitative real-time PCR and determined tyrosinase activity and melanin content at 24, 48, and 72 hours after transfection. Results showed that siNM_011661_001 was the most efficient small interfering RNA sequence in suppressing tyrosinase mRNA expression, and cells transfected with this sequence showed lower tyrosinase activity. Moreover, intravitreous injection of siNM_011661_001 in C57BL/6 mice induced an efficient and stable gene-specific inhibition of expression at the posttranscriptional level.

## 1. Introduction

Tyrosinase mutation can induce human diseases, such as oculocutaneous albinism. Melanin is a heterogeneous polymer produced and concentrated within the melanosomes of the melanocytes, and tyrosinase is a key rate-limiting enzyme for melanin biosynthesis. Tyrosinase catalyzes three steps in melanin biosynthesis: the hydroxylation of tyrosine to 3, 4-dihydroxyphenylalanine (DOPA), the oxidation of DOPA to DOPA-quinone, and the oxidation of 5, 6-dihydroxyindole to indolequinone. Because of its central role in melanogenesis, tyrosinase is a key target for the development of new inhibitors [1–3].

In the present study, we aimed to directly regulate tyrosinase expression using RNA interference (RNAi). We screened small interfering RNAs (siRNAs) corresponding to the tyrosinase sequence using the mouse melanoma cell line B16 and then delivered the most effective sequence into C57BL/6 mice to study its effect in vivo.

## 2. Materials and Methods 

### 2.1. Cell Culture

B16 mouse melanoma cells (Shanghai Institute of Cell Biology, Chinese Academy of Sciences) were cultured in DMEM (GIBCO, USA), supplemented with 10% (v/v) fetal bovine serum (Hangzhou Sijiqing Biological Engineering Materials Co., Ltd., China) and 2 mmol/L glutamine (AMRESCO, USA) at 37°C in a humidified atmosphere with 5% CO_2_ [3, 4]. The B16 cells were trypsinized and centrifuged at 1,500 rpm for 5 minutes and then resuspended in DMEM culture medium and seeded in a six-well plate (Corning, USA) at 2 × 10^5^ per well and 96-well plate (Corning) at 1 × 10^4^ per well. Cells were cultured for 12 hours and transfected when cells were at 30% to 50% confluence.

### 2.2. Mice

C57BL/6 mice were purchased from the Laboratory Animal Center at Sun Yat-sen University in China and were handled in accordance with the Research in Vision and Ophthalmology guidelines for the use of animals in research (animal quality certificate, 0028619; animal experiment license, SYXK [Guangdong] 2005-0058).

### 2.3. siRNA Design and Transfection

Twelve hours after seeding, cells were transfected using Lipofectamine 2000 (Invitrogen, USA) according to the manufacturer's instructions. siRNAs were prepared by serial dilution (10–60 nmol/L) in Opti-MEM I medium (GIBCO) to determine the optimal transfection concentration. The optimal transfection concentration was defined as the minimum concentration of siRNAs that can produce the highest fluorescence intensity. The siRNA-targeting tyrosinase mRNA was designed by the Dharmacon online siRNA design tool (http://www.dharmacon.com/HomePage.aspx) and synthesized by RuiBo Biotechnology Co., Ltd., China. The siRNA sequences against tyrosinase were as follows: siNM_011661_001 target sequence, AAGCGAGTCTTGATTAGAA; sense (5′-3′), AAGCGAGUCUUGAUUAGAA(dTdT) ; antisense (3′-5′), (dTdT)UUCGCUCAGAACUAAUCUU; siNM_ 011661_002 target sequence, ACAATGCCTTACATATCTT; sense, ACAAUGCCUUACAUAUCUU (dTdT); antisense, (dTdT)UGUUACGGAAUGUAUAGAA; and siNM_011661_003 target sequence, TCATACCGCTCTATAGAAA; sense, UCAUACCGCUCUAUAGAAA(dTdT); antisense, (dTdT) AGUAUGGCGAGAUAUCUUU. The siRNAs were all 3′-hydroxymethylation modified.

The siRNAs were diluted to the optimal concentration according to a previous description. A Lipofectamine 2000-containing negative siRNA (green fluorescence protein mRNA sequence) group and a blank group (Lipofectamine solution alone) were used as controls. After transfection, the cells were harvested at three different time points (24, 48, and 72 h) to determine the mRNA expression, melanin content, and tyrosinase activity for each treatment. The grouping information is as follows: group1 (siNM_011661_001), group2 (siNM_011661_002), group3 (siNM_011661_003), negative control group (negative siRNA), blank group (Lipofectamine).

### 2.4. Quantitative Real-Time PCR

Tyrosinase mRNA in siRNA-treated B16 cells was quantitatively analyzed using quantitative real-time PCR (qRT-PCR). Total RNA extraction from B16 cells was done as previously described [5]. cDNA was synthesized with random primers using no more than 500 ng of total RNA in each 10-*μ*L reaction according to the manufacturer's instruction (TaKaRa, Japan). SYBR Green qRT-PCR were done in a Real-Time Detection System (ABI7000, USA) according to the manufacturer's instructions (TaKaRa, Japan) using the comparative cycle threshold method. *β*-actin mRNA was used as the internal control. Thermal cycler conditions were 95°C for 10 seconds followed by 40 cycles of 5 seconds each at 95°C and 31 seconds at 60°C. The primer pairs were designed for tyrosinase and *β*-actin using Primer 5.0 Express Software as follows: tyrosinase forward primer, 5′-GCCCAGCATCCTTCTTC-3′; tyrosinase reverse primer, 5′-TAGTGGTCCCTCAGGTGTTC-  3′; *β*-actin forward primer, 5′-CAGCCTTCCTTCTTGGGTAT-  3′; and *β*-actin reverse primer, 5′-TGGCATAGAGGTCTTTACGG-  3′.

### 2.5. Tyrosinase Activity Assay

Tyrosinase activity was assessed by determining the catalysis of L-DOPA to dopachrome, which has an absorption peak at 490 nm. In this study, the inhibitory action of siRNAs on tyrosinase was assayed as described previously [4, 6, 7] with slight modifications. Briefly, B16 cells were seeded at a density of 1 × 104 cells per well in 96-well plates. After 12 hours, the cells were treated with different siRNAs and cultured for 24 to 72 hours at 37°C The adherent cells were washed three times with PBS and then lysed in sodium phosphate buffer (0.1 mol/L, pH 6.8) containing 1% Triton X-100. Then, 10 *μ*L L-DOPA (1%; ICN, China) was added, allowed to react, and incubated for 2 hours at 37°C. Absorbance was measured at 490 nm using an enzyme-linked immunosorbent assay reader (PowerWave XS, Bio-Tek, USA). These experiments were done in triplicate. 

### 2.6. Assay of Melanin Content

The melanin content was determined as described previously [4, 7–9] by alkaline lysis with minor modifications. After transfection, adherent cells (1 × 10^6^) were washed with PBS, detached by trypsinization, collected in a test tube, and then washed twice with PBS. The cells were resuspended in 1 mol/L NaOH, incubated at 80°C for 30 minutes, and then centrifuged at 1,500 rpm for 5 minutes. The absorbance of the supernatant was measured at 475 nm and compared with the standard curve of the serial dilution of standard melanin (Sigma, USA). The melanin content was expressed as microgram per 1 × 10^6^ cells. The detection was done in triplicate.

### 2.7. Analysis of Retinal Tyrosinase mRNA in siNM_011661_001 Treated Mice

Mice received general anesthesia by the i.p. injection of chloral hydrate (0.008 mL/kg) and local anesthesia with 2% pontocaine. With the guidance of an operating microscope (OLYMPUS, Japan), two groups of mice were given an intravitreous injection of 2.5 *μ*L PBS containing either 0.65 or 1.3 nmol siNM_011661_001, and 2.5 *μ*L PBS was injected as control. The detection was done in triplicate in every group. Twenty-four hours after injection, total RNA extraction (QIAGEN, Germany) was done from the retina of C57BL/6 mice as described previously [5] and analyzed with qRT-PCR. 

### 2.8. Statistical Analysis

Data were expressed as mean ± SD. ANOVA was used to compare the differences between the groups using SPSS 13.0 for Windows (Chicago, USA). *P* < .05 was considered statistically significant. 

## 3. Results

### 3.1. Cell Morphology

**Figure 1:**
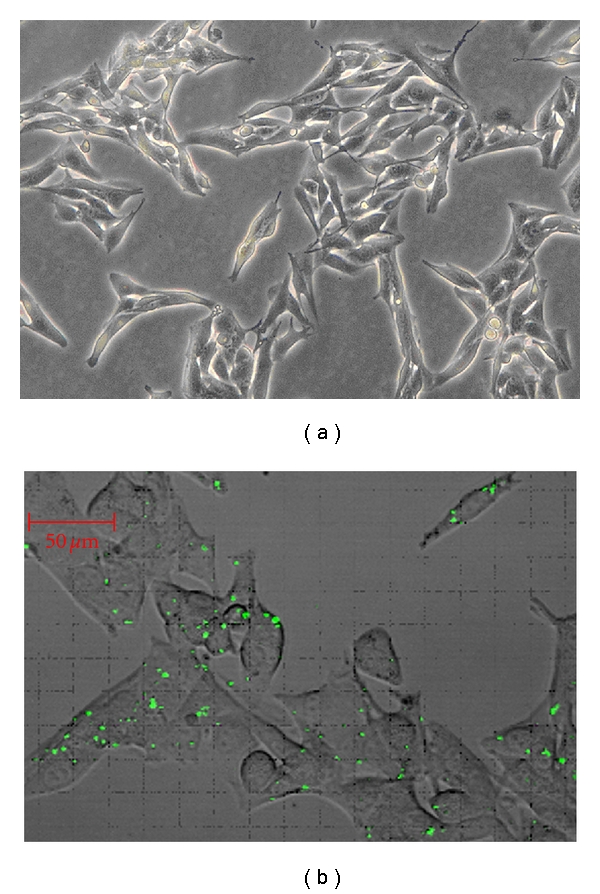
Light micrograph showing the morphology of B16 mouse melanoma cells before transfection. Original magnification, ×  100. Determination of the optimal siRNA concentration using the fluorescently labeled (FAM) negative control siRNA Original magnification, ×  200. The siRNA concentration used in these experiments was 40 nmol/L.

Before transfection, most cells appeared fusiform, and some cells were reticular at low density. The cells in the division phase showed a clear outline, an obvious boundary between cells, and a fine refractive index (Figure 1), but after transfection, the cells swelled and the boundary between them became indistinct.

### 3.2. Determination of the Optimal Transfection Concentration

Twenty-four hours after transfection, the distribution of intracellular fluorescence was sporadic in cells treated with 10, 20, 30, and 60 nmol/L siRNA. However, intracellular fluorescence was widespread, and the intensity was strengthened in cells transfected with 40 and 50 nmol/L siRNA (Figure 1). In this study, we achieved the best transfection efficacy with the lowest Lipofectamine 2000 (0.2 *μ*L per cell in a 96-well plate; 4 *μ*L per cell in a 6-well plate) and siRNA concentrations (40 nmol/L). Thus, we used 40 nmol/L in this study.

### 3.3. mRNA Expression

**Figure 2:**
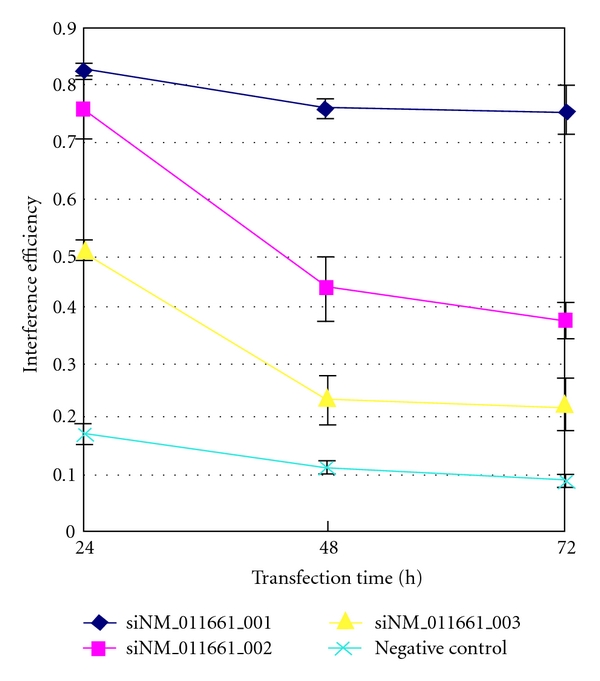
The efficacy of each siRNA in suppressing mRNA at different time points. Interference efficiency of siNM_011661_001 (blue), siNM_011661_002 (pink), siNM_011661_003 (yellow), and negative siRNA (green) at 24, 48, and 72 hours after transfection. Experiments were done in triplicate. Results are presented with respect to the Lipofectamine-treated group, which was set at 100%.

We evaluated the efficiency of each sequence to suppress tyrosinase expression at 24, 48, and 72 hours after transfection. The results were 82.81%, 76.19%, and 75.46% for siNM_011661_001; 75.75%, 43.49%, and 37.86% for siNM_011661_002; 50.54%, 23.66%, and 22.42% for siNM_011661_003; 17.22%, 11.34%, and 8.68% for the negative control sequence, respectively (Figure 2). The efficiency of the three fragments decreased gradually from 24 to 72 hours after transfection. The statistical results showed that the siRNA sequence siNM_011661_001 was the most effective in suppressing tyrosinase expression (*F * = 298.566 and *P* < .05 at 24 h; *F * = 179.217 and *P * < .05 at 48 h; *F * = 188.564 and *P* < .05 at 72 h). 

### 3.4. Tyrosinase Activity

**Figure 3:**
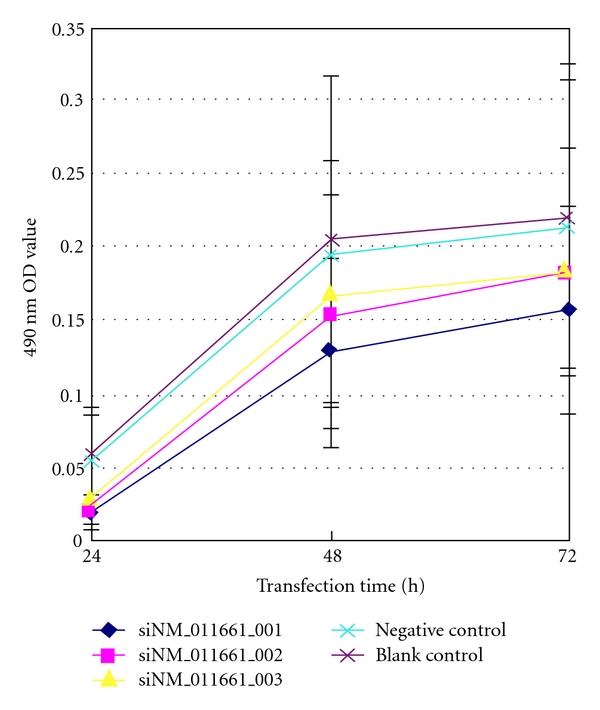
Detection of tyrosinase activity (OD value) at different time points. Absorbance was measured at 490 nm to determine dopachrome production in B16 cells treated with siNM_011661_001 (blue), siNM_011661_002 (pink), siNM_011661_003 (yellow), negative siRNA (green), and Lipofectamine (brown).

We evaluated reductions in tyrosinase activity in the transfected cells at 24, 48, and 72 hours after transfection. Tyrosinase activity was reduced by 64.73%, 40.42%, and 28.74% for siNM_011661_001; 63.37%, 25.06%, and 17.33% for siNM_011661_002; 48.16%, 18.33%, and 16.65% for siNM_011661_003; 6.44%, 4.90%, and 3.56% for the negative control sequence, respectively (Figure 3). The tyrosinase suppression decreased with time for each siRNA sequence, which was similar to the suppression at the mRNA level. Overall, siNM_011661_001 had the most powerful interference efficiency. After 24 hours, there was no significant difference between siNM_011661_001 and siNM_011661_002 (*P * > .05), whereas significant differences were observed among the other groups (*F * = 58.485; *P * < .05). At 48 hours, significant differences were observed among the groups (*F * = 160.208; *P * < .05). At 72 hours, no significant difference was found between siNM_011661_002 and siNM_011661_003, whereas significant differences were observed between the other groups (*F* = 117.372; *P* < .05). These results supported previous findings that siNM_011661_001 most effectively suppressed tyrosinase expression.

### 3.5. Melanin Content

**Figure 4:**
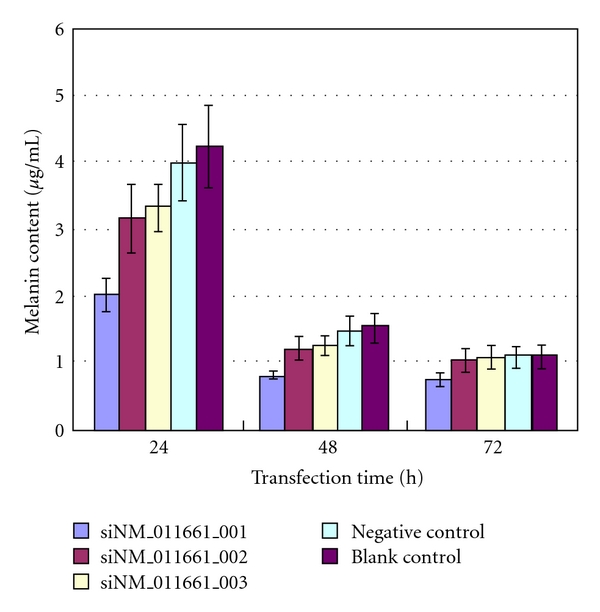
Detection of melanin content at different time points. The absorbance was measured at 475 nm and compared with the standard curve made by the serial dilution of standard melanin. The melanin content was expressed as microgram per 1 × 106 cells. Melanin content was determined in B16 cells treated with siNM_011661_001 (blue), siNM_011661_002 (pink), siNM_011661_003 (yellow), negative siRNA (green), and Lipofectamine (brown) at 24, 48, and 72 hours after transfection. Experiments were done in triplicate.

The decrease in melanin content in each group was evaluated at days 1, 2, and 3. The percent reduction in melanin was 52.53%, 48.55%, and 32.73% for siNM_011661_001; 25.59%, 21.61%, and 6.15% for siNM_011661_002; 21.01%, 18.79%, and 1.70% for siNM_011661_003; 5.53%, 3.58%, and 1.00% for the negative control sequence, respectively (Figure 4). After 48 hours, the melanin content and interference efficiency of siNM_011661_002 and siNM_011661_003 were not significantly different. However, after 72 hours, cells transfected with siNM_011661_001 contained significantly less melanin than the other groups (*P *<.05), whereas comparisons between other groups were not significantly different.

### 3.6. Measurement of mRNA Expression of Tyrosinase In Vivo

Because siNM_011661_001 most effectively reduced mRNA expression of tyrosinase in B16 cells, we evaluated its effect in C57BL/6 mice. To determine the change in tyrosinase mRNA levels, qRT-PCR was carried out using tyrosinase-specific primers and total RNA isolated from retinas (Table 1). Negative interference sequence and liposome (blank group) have nonspecific interference effect during our experiment.

## 4. Discussions

All three siRNA sequences that we screened suppressed tyrosinase expression, but siNM_011661_001 was most effective, exerting the maximum effect at 24 hours after transfection. It is presumed that tyrosinase mRNA possesses a special functional structure that is most similar to siNM_011661_001. The data presented here suggests a crucial role for siRNA structure in RNAi [8, 10].

In this study, the suppressive effects on melanogenesis in B16 cell line peaked at 24 hours, suggesting that siRNA was active for 24 hours after transfection and then was gradually degraded. However, we used modified siRNA, which can enhance stability and reduce the potential for off-target effects. Sequences of siRNA for a target gene do not perform equally, but significant progress has been made in defining sequence features that contribute to siRNA potency. Low internal stability at the 5′ terminus of antisense siRNA is an important prerequisite for gene silencing. Excessive stability can result in double-strand unwinding, but inferior stability can reduce the affinity of siRNA to its mRNA target [11]. 

In the present study, we noted an interesting phenomenon: siNM_011661_001 transfection resulted in the considerable suppression of tyrosinase mRNA compared with other control siRNAs. Further experiments are needed to support this finding.

Furthermore, the negative controls (siRNA sequence and Lipofectamine transfection reagent) were observed to slightly reduce melanin content with time (Figure 4), which was similar to the effect of siNM_011661_002 and siNM_011661_003 72 hours after transfection. This phenomenon also appeared at the level of gene expression and tyrosinase activity, suggesting nonspecific RNAi activity. Thus, negative control siRNA should be selected carefully.

In addition to showing that siNM_011661_001 is the most potent siRNA sequence against tyrosinase in B16 cells, we assessed the effect of this siRNA sequence on tyrosinase expression in C57BL/6 mice. One of the important factors for RNAi effectiveness in vivo may be the delivery and transfection efficiency of siRNAs. In mammalian cells, siRNA has been administered locally or systemically, and the efficiency of RNAi delivery systems is still relatively low. Previous studies showed that the systemic delivery of siRNAs by tail vein injection does not deliver siRNAs efficiently to their target areas because siRNA molecules are easily trapped in nonspecific organs. We thought that the optimal efficacy of siRNA targeting pigmentary epithelium would be largely deprived via intravenous injection, because the nutrient vessel supporting pigmentary epithelium belongs to distal circulation vessel. What is more, the synthetic siRNA pieces are heterogenous to mice; appearance of these heterogenous pieces in circulation may potentially induce severe immune lesion to mice. In contrast, intravitreous treatment may have its advantage to overcome these problems, because vitreous space has the immediate proximity to retina, the small molecular weight of siRNA may be efficiently delivered to retina and pigment epithelium through posterior vitreous membrane and thus have direct effects on target tissues. In addition, the blood-ocular barrier may function to prevent the entry of intravitreously injected siRNA into circulation. In summary, intravitreous administration may take many advantages beyond intravenous injection, due to efficient and direct delivery of siRNA pieces to target tissues combined with reduced risk of inducing side effects. After considering these factors collectively, we chose intravitreous injection throughout this work. We also believe that this method would provide a new approach to ocular albinism mouse model preparation. In contrast, our results showed that siRNA administered locally had significant inhibitory effects on the retina. It strongly suggests that siNM_011661_001 can be delivered effectively in vivo and may also be delivered to other tissues such as skin and hair.

In summary, we have shown that siNM_011661_001-targeting tyrosinase is the most potent sequence in vitro and in vivo. RNAi is a new tool for gene silencing using antisense nucleic acids [12] and gene targeting [13]. In addition, it gives us a new method to use in the development of pigmentation disease (e.g., ocular albinism) animal models and treatments. Furthermore, this technique may be useful in the study of downstream factors of tyrosinase.

## Figures and Tables

**Table 1:** The tyrosinase mRNA levels in mice receiving different dosages of siNM_011661_001.

Dosage	0.65 nmol siNM_011661_001	1.3 nmol siNM_011661_001
Interference efficiency*	25.25% ± 0.0355	59.85% ± 0.0276

*Twenty-four hours after injection, qRT-PCR showed that the tyrosinase levels in the retinas of mice treated with 0.65 and 1.3 nmol siNM_011661_001 siRNA were reduced by 25.25% ± 0.0355 and 59.85% ± 0.0276 (*P *<  .05), respectively, compared with retinas of eyes injected with PBS.

## References

[1] Schallreuter KU, Kothari S, Chavan B, Spencer JD (2008). Regulation of melanogenesis-controversies and new concepts. *Experimental Dermatology*.

[2] Simon JD, Peles D, Wakamatsu K, Ito S (2009). Current challenges in understanding melanogenesis: bridging chemistry, biological control, morphology, and function. *Pigment Cell and Melanoma Research*.

[3] Yang J-Y, Koo J-H, Song Y-G (2006). Stimulation of melanogenesis by scoparone in B16 melanoma cells. *Acta Pharmacologica Sinica*.

[4] Kim YJ, No JK, Lee JS, Kim MS, Chung HY (2006). Antimelanogenic activity of 3,4-dihydroxyacetophenone: inhibition of tyrosinase and MITF. *Bioscience, Biotechnology and Biochemistry*.

[5] Dörrie J, Wellner V, Kämpgen E, Schuler G, Schaft N (2006). An improved method for RNA isolation and removal of melanin contamination from melanoma tissue: implications for tumor antigen detection and amplification. *Journal of Immunological Methods*.

[6] Nakajima M, Shinoda I, Fukuwatari Y, Hayasawa H (1998). Arbutin increases the pigmentation of cultured human melanocytes through mechanisms other than the induction of tyrosinase activity. *Pigment Cell Research*.

[7] Ando H, Funasaka Y, Oka M (1999). Possible involvement of proteolytic degradation of tyrosinase in the regulatory effect of fatty acids on melanogenesis. *Journal of Lipid Research*.

[8] Peek AS, Behlke MA (2007). Design of active small interfering RNAs. *Current Opinion in Molecular Therapeutics*.

[9] Ishikawa M, Kawase I, Ishii F (2007). Combination of amino acids reduces pigmentation in B16F0 melanoma cells. *Biological and Pharmaceutical Bulletin*.

[10] Bradáč I, Svobodová Vařeková R, Wacenovsky M, Škrdla M, Plchút M, Polčík M (2007). siRNA selection criteria-Statistical analyses of applicability and significance. *Biochemical and Biophysical Research Communications*.

[11] Reynolds A, Leake D, Boese Q, Scaringe S, Marshall WS, Khvorova A (2004). Rational siRNA design for RNA interference. *Nature Biotechnology*.

[12] Higashi Y, Asanuma M, Miyazaki I, Ogawa N (2000). Inhibition of tyrosinase reduces cell viability in catecholaminergic neuronal cells. *Journal of Neurochemistry*.

[13] Boonanuntanasarn S, Yoshizaki G, Iwai K, Takeuchi T (2004). Molecular cloning, gene expression in albino mutants and gene knockdown studies of tyrosinase mRNA in rainbow trout. *Pigment Cell Research*.

